# Digital roots: harnessing digital platforms in advancing traditional and complementary medicine for cancer care in Sub-Saharan Africa

**DOI:** 10.3332/ecancer.2024.ed132

**Published:** 2024-03-14

**Authors:** Dang Nguyen, Nityanand Jain, Akul Gupta, Cameron Sabet, Simar S Bajaj, Saloni Patel, Heath Rutledge-Jukes, Michael J Diaz, Bao-Tran Do Le, Olivia A Hamati, Muhammad Mustafa, Saso Krstovski, Twalib Ngoma, Wilfred Ngwa

**Affiliations:** 1 Massachusetts General Hospital, Corrigan Minehan Heart Center, Harvard Medical School, Boston, MA, USA; 2 Judy Genshaft Honors College, University of South Florida, Tampa, FL, USA; 3 Faculty of Medicine, Riga Stradinš University, 16 Dzirciema street, Riga, Latvia; 4 Georgetown University School of Medicine, Washington, DC, USA; 5 Harvard University, Cambridge, MA, USA; 6 Johns Hopkins University School of Medicine, Baltimore, MD, USA; 7 Washington University in St. Louis School of Medicine, Saint Louis, MO, USA; 8 College of Medicine, University of Florida, Gainesville, FL, USA; 9 Department of Otolaryngology, Harvard Medical School, Boston, MA, USA; 10 University of South Florida, Tampa, FL, USA; 11 Department of Biological Sciences, University of Michigan, Ann Arbor, MI, USA; 12 Department of Clinical Oncology, Muhimbili University of Health and Allied Sciences, Dar Es Salaam, Tanzania; 13 Brigham and Women’s Hospital, Dana-Farber Cancer Institute, Harvard Medical School, Boston, MA, USA; 14 Sidney Kimmel Comprehensive Cancer Center, Johns Hopkins University School of Medicine, Baltimore, MD, USA; * Authors contributed equally (co-first authors)

**Keywords:** Sub-Saharan Africa, traditional and complementary medicine, digital health, cancer

## Abstract

The rise in cancer rates in Sub-Saharan Africa (SSA), combined with limited access to Western pharmaceuticals, has sparked growing adoption of traditional and complementary medicine (T&CM) for cancer treatment in the region. However, many challenges exist, including the lack of reliable evidence-based research on these products, scarcity of standardized documentation as part of cancer registries, limited physician expertise, and negative effects on mortality. Nonetheless, herbal medicines also present opportunities for further research, development, and stakeholder education, potentially benefiting the regional healthcare systems in SSA countries and global health as whole. Recent trends highlight the willingness of patients to use mobile-based applications that provide accurate information on herbal therapeutics, reflecting the increasing adoption of internet and smart/mobile phone services in SSA. To maximize the potential benefits of traditional and complementary medicine, it is necessary to bridge the trust gap between the public, local practitioners, and Western healthcare providers. Sustained funding and policy support are needed to complement these initiatives. Our preliminary survey hopes to inspire the community and policymakers to embrace innovative solutions, fostering a forward-looking approach to cancer care in SSA.

It is estimated that 80% of the world’s population relies on herbal medicines as a source of primary care — a figure that is expected to rise as acceptance and awareness increase beyond developing countries [1]. Reflecting shifting public interest, T&CM herbs have not only become more widely available in supermarkets, pharmacies, and e-stores, but have also been included in Western pharmacopeias. The 2017 European Pharmacopoeia, a legally binding document in 39 countries, lists 219 herbal aggregates [2], an increase of 32 monographs from 2013. In Africa, where more than 5,000 botanical species are currently in therapeutic use, medicinal herbs are particularly diverse and commonly integrated into daily life for various health purposes.

Due to its equatorial geography, the African continent receives stronger ultraviolet rays. It has been suggested that this may result in higher levels of chemo-preventive bioactive metabolites in local flora than counterparts in the Northern Hemisphere [3]. Importantly, major anticancer drugs, including paclitaxel and arsenic trioxide, are derived from natural sources, and countries such as the United States, India, and China have made great strides in testing and registering potential herbs for cancer treatment [4]. Our recent *Lancet Oncology Commission Report* has emphasized the importance of considering the use of phytomedicines or medicinal plants in the development of National Cancer Control Plans (NCCPs) in sub-Saharan Africa (SSA) countries [5], a region that in the past 30 years alone, has witnessed doubling of cancer incidence rates. Alarmingly, our report also estimated that a record one million people in the region will die from cancer by 2030 [5], partially due to lack of appropriate screening and treatment access, as well as mistrust of Western medicine.

T&CM is widely practiced and trusted by local people, primarily due to religious folklore, ease of access, and lower cost [6]. An estimated one-third of cancer patients in the SSA region reported using herbal medicines for cancer treatment [7]. This prevalence presents us with both a challenge and an opportunity (Table 1). While it provides a common platform for discussion and engagement with local communities, it also highlights the need for sustained funding and reliable evidence-based research. Research has shown that T&CM, when combined with Western medicine, extends the reach of modern medicine while remaining cost-effective for patients [8]. These findings highlight the need for further research to maximize the benefits and minimize any negative effects arising from the practice of T&CM.

However, the limited expertise of T&CM providers and the risk of increased morbidity and mortality remains a significant challenge. This issue is exacerbated by the non-inclusion of T&CM practitioners in formal health systems and the relative neglect of herbal medicine in medical education curricula [9]. We believe that bridging the trust gap between stakeholders, including the public, local practitioners, and Western health care providers, is the first step in this direction. Familiarization with local T&CM practices would likely increase health professionals’ awareness of their use, side effects, and drug interactions, thereby enabling rapid identification of unethical and unscientific practices [10]. It would also enable clinicians to provide guidance on treatment options by reducing the stigma and negative connotations associated with this practice.

A second pressing issue is the urgent need to summarize the extent of herbal use for cancer treatment in African countries, especially given that much of the published literature is available in grey journals, limiting searchability and trustworthiness. Although some studies have attempted to compare the benefits and cost-effectiveness of T&CM and Western medicine, small homogeneous sampling and conflicting results often limit generalizability. The establishment of dedicated and comprehensive cancer registries has been proposed to overcome this problem [11], but we believe that such registries would have limited practicality because they primarily collect data from hospitals, perpetuating the Western medical model, and exclude data from traditional caregivers.

In this regard, we herein report our experience with a cohort of 23 prostate cancer patients at the Ocean Road Cancer Institute in Dar es Salaam, Tanzania (Table 2). All patients reported to have health insurance, with some being regular users of T&CM as an adjunct to cancer therapy. When specifically asked, most used herbs for symptomatic relief, with a few using them for erectile dysfunction, hypertension, and diabetes. None but one reported side effects after taking herbal medicines.

While an overwhelming majority agreed that herbal medicines were cheaper than prescription medicines, they also believed that they were less effective. There are several plausible explanations for this observation. First, doctors in Tanzania often discourage the use of herbal medicines because of uncertainty about drug-herb interactions. Second, doctors who prescribe herbal medicines tend to be distrusted. In the words of one patient, “*(The) herbal clinic here doesn’t investigate what my medical condition is. They just look at the symptoms and give (me) medications that I don’t trust.*” In addition, patients believe that doctors often use their beliefs to coerce them by prescribing expensive herbal medicines that are not covered by insurance.

The growing reliance on T&CM in SSA underscores the need for easily accessible, verifiable information on herbal therapeutics. Given the promise of digital health platforms in disseminating accurate medical knowledge and improving medical literacy, we pioneered the development of a mobile application (TeleCatalyst [12]) to provide a comprehensive compendium of herbs, elucidating aspects such as common nomenclature, statistical data, identification methods, therapeutic virtues/evidence, potential adverse effects, and their integration with conventional medical treatments. In our cohort of prostate cancer patients, it was encouraging to note that 70% (16/23) of patients were willing to use a mobile application that provided relevant information on the medical use of recognized herbal remedies. Considering the average age of participants was over 65 years, this is even more welcoming. Noticeably, for the remaining seven patients (30%), lack of data reliability and physician approval were the primary reasons for refusal, rather than a specific lack of interest in mobile applications or technology. These findings reflect the rapid adoption of internet and smartphone services across Africa, which according to the Groupe Speciale Mobile Association (GSMA) will exceed 600 million active mobile subscribers by 2025, surpassing Europe and North America.

In resource-limited settings, especially in developing countries where the nearest health center may not be easily accessible, telemedicine has tremendous potential. AYUSH digital T&CM health initiatives in India have provided a blueprint for reaching over a billion people [13]. Although an initial lump-sum investment by public authorities may be a barrier to rapid adoption, in the long run these costs could be recouped within the first few years of implementation. A recent U.S. analysis of mid-aged oncology patients estimated savings of $150 to $200 per patient per visit by implementing telehealth strategies [14]. Promises of reducing the oncological burden on the national budget could encourage local governments to subsidize and fund the initial investment in hospitals.

We believe our preliminary survey will encourage the community and policy makers to move towards a more sustainable and forward-looking application of T&CM, particularly in cancer care, through implementing telemedical solutions. However, barriers to the adoption remain in SSA, including broadband latency and unreliable electricity connections. There is also an urgent need for patient education and clear definition of medical terminology within the digital application to avoid misinterpretation of jargon. To effectively drive this initiative, government support and new policy and regulatory frameworks are essential.

## Conclusions

The convergence of traditional medicine and modern health care can help address gaps in cancer screening, treatment modalities, and access to care in SSA, but success requires concerted efforts given challenges. Given the limitations of existing studies and cancer registries, there is an urgent need to summarize the extent of herbal use for cancer treatment in African countries. Our experience with a cohort of prostate cancer patients in Tanzania demonstrates patient’s interest in digital health platforms to disseminate accurate information, improve medical literacy, and facilitate informed decision-making among patients. Telemedicine reflects a shifting healthcare landscape, and its integration can improve the accessibility, reliability, and effectiveness of cancer care in SSA.

## Conflicts of interest

The author(s) declare that they have no conflict of interest.

## Funding

Not applicable.

## Competing interests

None.

## Figures and Tables

**Table 1.** Challenges and opportunities associated with adoption of T&CM in SSA region^†^.

Challenges	Opportunities
Inadequate and interrupted funding for research, monitoring, and development Limited national and regional strategic plans for policy implementation including inclusion of and collaboration with Traditional Health Practitioners Limited resources to conduct Phase-III clinical trials to collect and document scientific evidence Improper registration of intellectual property rights and preservation of indigenous knowledge Limited investments and initiatives in capacity-building, especially in collaboration with the private sector Weak dissemination of research results and information exchange with stakeholders and public Uncertainties in ensuring quality control due to genetic, environmental, and logistical factors Lack of technology for harvesting, cultivation, sanitation, storage, and preservation of medicinal extracts for extended shelf life Limited pharmacovigilance and challenges in prevention of adulteration and off-label resell Differences in regulatory requirements concerning classification of herbal medicines as a food, a functional food, a dietary supplement or an herbal medicine Lack of insurance coverage for the use and purchase of herbal medicines Late presentation of the patient to the healthcare system thereby leaving limited alternative referral pathways including palliative care	Formulation of national and regional regulatory policies and frameworks Production and collection of scientific evidence on safety, efficacy, and quality of T&CM Development of inventories and monographs on medicinal plants and herbal pharmacopoeias Inclusion of T&CM in training curricula of health professionals Continuing education and skills development programs for Traditional Health Practitioners including their integration in national healthcare systems Employment opportunities by promoting local cultivation, production, and packaging of medicinal plants Registration of international intellectual property rights and subsequent commercialization Encourage sustainable use of traditional-knowledge-related biodiversity Equitable share the benefits arising from the commercial use of traditional knowledge including price regulation for sustainable living Discovery of new active compounds from herbs or new indications for existing herbal medications Bridging the trust gap so as to increase healthcare seeking behavior and early screening, prevention, and presentation Boosting of national economies, health situation, and literacy by integrating information and communication technology with one health approach

† Adapted from The African Health Monitor: African Traditional Medicine Day, 31 August 2010 (Special Issue). World Health Organization Regional Office for Africa. Available from https://www.afro.who.int/sites/default/files/2017-06/ahm-special-issue-14.pdf?fbclid=IwAR2yQI7PIWL5k9pXKkAPsPA1tJFVjAvHBNWDO-KjqPE_gEf-GCQhLGGo9sg (accessed 03/02/2024).

**Table 2.** Results of interviews from 23 prostate cancer patients.

Questionnaire	Agree (%)	Disagree (%)
I have used herbs for medical treatment at least once.	12 (52.2)	11 (47.8)
I believe herbal medications is better than prescription medicine.	3 (13.0)	20 (87.0)
I believe herbal medications are less expensive than prescription medicine.	18 (78.3)	5 (21.7)
I regularly use herbal medications.	9 (39.1)	14 (61.9)
I have experienced side effects after using herbal medications.	1 (4.3)	22 (95.7)
I would use a mobile app with information on herbs for my medical treatment.	16 (69.6)	7 (30.4)
